# Development and validation of an explainable machine learning model for risk stratification in patients with clinically suspected acute pulmonary embolism: retrospective study

**DOI:** 10.1016/j.clinsp.2026.101138

**Published:** 2026-09-10

**Authors:** Qingming Li, Huijun Qin, Jian Xu

**Affiliations:** Department of Clinical Laboratory, Dazhou Central Hospital, Tongchuan District, Dazhou, Sichuan Province, China

**Keywords:** Acute pulmonary embolism, Risk prediction model, Machine learning, Shapley additive explanations (SHAP)

## Abstract

**Background:**

Acute Pulmonary Embolism (APE) is a life-threatening cardiovascular emergency. Computed Tomography Pulmonary Angiography (CTPA) serves as the diagnostic gold standard but is associated with risks of overuse and radiation exposure.

**Objective:**

To develop and validate an explainable machine learning model for predicting the likelihood of APE in clinically suspected patients, aiming to optimize CTPA decision-making.

**Methods:**

This study retrospectively enrolled 649 patients who underwent CTPA for suspected APE based on clinical symptoms, signs, or elevated D-dimer levels. Seventy-seven clinical variables were collected. Least Absolute Shrinkage and Selection Operator (LASSO) regression was used for initial feature screening, followed by multivariate logistic regression to identify independent predictors. Eight machine learning algorithms were compared to select the optimal model. The Shapley Additive exPlanations (SHAP) framework was applied for model interpretability analysis.

**Results:**

The model demonstrated stable performance in both training and validation sets (training set AUC: 0.865±0.003; validation set AUC: 0.843±0.038). In the independent test set, the logistic regression model achieved the best performance (AUC = 0.793). Thirteen key predictors were identified, including age, lactate, fibrin degradation products, and others. SHAP analysis visually illustrated the contribution of each feature to risk prediction.

**Conclusions:**

The developed APE risk prediction model, based on logistic regression and SHAP interpretability, exhibits good discriminative ability and transparency. It shows potential for individualized risk stratification and optimization of CTPA utilization in clinical decision-making.

## Highlights

• A machine learning model predicts acute pulmonary embolism risk in suspected patients.
• LASSO and logistic regression identified key clinical predictors from 77 variables.
• Eight ML algorithms were compared to select the optimal predictive model.
• SHAP framework provides interpretable explanations for individual risk predictions.
• The model aims to reduce unnecessary CTPA overuse and radiation exposure.

## Introduction

Acute Pulmonary Embolism (PE), a critical medical emergency, arises from the dislodgement of thrombi originating in the deep venous system or right heart chambers, which subsequently embolize to and obstruct the pulmonary arteries or their branches. As the most severe manifestation of venous thromboembolism, PE carries a significant mortality risk if not promptly diagnosed and effectively treated. Epidemiological data identify PE as the third leading cause of cardiovascular death worldwide, following only stroke and acute myocardial infarction.^1^ Despite advancements in management, high-risk patients presenting with hemodynamic instability or cardiac arrest continue to face mortality rates exceeding 40%, primarily due to acute right ventricular failure.^2^ Computed Tomography Pulmonary Angiography (CTPA) remains the gold standard for PE diagnosis.^3^ However, its widespread application presents clinical challenges, including overutilization, radiation exposure, and potential delays in diagnostic workflows.^4^ While traditional clinical decision rules, such as the Wells criteria, aid in rationalizing CTPA use, their reliance on a limited set of clinical variables constrains their discriminatory power in complex cases.^5^ Although biomarkers like high-sensitivity cardiac troponin, B-type natriuretic peptide (and its precursor NT-proBNP), and D-dimer correlate with PE severity,^6^^,^^7^ the predictive capacity of individual markers remains insufficient, underscoring the need for tools that integrate multi-dimensional clinical data for precise risk assessment.^8^

Machine Learning (ML) offers a powerful solution for analyzing high-dimensional clinical datasets and capturing complex, non-linear relationships, demonstrating considerable promise in disease prediction.^9^^,^^10^ A major barrier to the clinical adoption of conventional ML models is their “black-box” nature, which limits interpretability and erodes clinician trust.^11^ Explainable Artificial Intelligence (XAI) techniques, particularly the Shapley Additive exPlanations (SHAP) framework, address this critical gap by quantifying the contribution of each input feature to model predictions, thereby rendering the decision-making process transparent and clinically actionable.^12^ To bridge this translational gap, we developed and validated a high-performance, interpretable prediction model for PE in clinically suspected patients. Our approach integrates a comprehensive panel of clinical variables spanning demographics, blood gas analysis, cardiac injury markers, and coagulation profiles. We employed Least Absolute Shrinkage and Selection Operator (LASSO) regression for feature selection and compared multiple ML algorithms, ultimately identifying a Logistic Regression model as optimal. By incorporating the SHAP framework for both global and individualized model interpretation, this study aims to provide a novel clinical decision-support tool that combines high predictive accuracy with essential explainability, facilitating optimized CTPA utilization and enabling earlier, more precise identification of acute PE.

## Materials

### Study population

A retrospective cross-sectional study was conducted. We consecutively included 649 patients who underwent Computed Tomography Pulmonary Angiography (CTPA) for suspected acute pulmonary embolism at Dazhou Central Hospital, Sichuan Province, between January 1, 2022, and October 1, 2025. Suspicion was based on clinical presentation (chest pain, dyspnea, hemoptysis) or elevated D-dimer levels. To ensure clinical validity and reflect a realistic pre-test prediction scenario, all predictor variables were strictly limited to data available prior to CTPA, with the time window for collection restricted to the 24 h preceding the examination. Clinical, laboratory, and imaging data were systematically extracted from the hospital's structured Electronic Medical Records (EMR) and Laboratory Information Systems (LIS). The dataset was first partitioned into a training set and a testing set using stratified sampling. All preprocessing, including missing data imputation and feature selection, was subsequently performed exclusively on the training set using cross-validation, with the testing set reserved solely for final unbiased model evaluation. This sequential workflow was implemented to prevent data leakage and avoid overestimation of model performance.

### Inclusion criteria

Patients were included if they met all the following criteria: 1) Clinical suspicion of APE: Presence of at least one clinical manifestation or laboratory abnormality suggestive of APE, including: new-onset or worsening dyspnea, chest pain, hemoptysis, syncope, or palpitations during hospitalization; respiratory rate > 20 breaths/min; tachycardia (heart rate > 100 beats/min); decreased oxygen saturation (SpO₂ < 94% on room air); or elevated plasma D-dimer level. 2) Diagnostic evaluation: Underwent Computed Tomography Pulmonary Angiography (CTPA) following clinical assessment for definitive diagnosis. 3) Data completeness: Complete data for all study parameters (or data suitable for reasonable imputation) were available within 24-h before or after the CTPA examination.

### Exclusion criteria

Patients were excluded if they met any of the following conditions: 1) Pregnancy or lactation. 2) End-stage renal disease requiring regular dialysis or estimated Glomerular Filtration Rate (eGFR) < 15 mL/min/1.73 m^2^, due to potential significant effects on biochemical and coagulation parameters. 3) Diagnosis of Chronic Thromboembolic Pulmonary Hypertension (CTEPH). 4) Patients with hemodynamic instability (systolic blood pressure < 90 mmHg, requirement of vasopressors, or cardiac arrest) were excluded from model development, as current clinical guidelines recommend immediate reperfusion therapy in such cases without awaiting CTPA or risk stratification scores. 5) Critical data missing, defined as > 30% missingness in key variables (e.g., outcome or core predictors).

## Methods

### Study grouping based on reference standard

All enrolled patients were categorized into two groups based on the gold-standard CTPA diagnosis: the Acute Pulmonary Embolism (APE) group (positive cases), comprising patients with a definitive CTPA-confirmed diagnosis of APE; and the Non-Acute Pulmonary Embolism (non-APE) group (negative cases), comprising patients in whom APE was excluded by CTPA.

This retrospective study was conducted in accordance with the ethical principles of the Declaration of Helsinki. This study received ethical approval from the Institutional Ethics Review Committee of Dazhou Central Hospital. (Approval n° 20,260,109). Informed consent was waived by the same committee because the study used fully anonymized historical patient data.

### Study variables

This study aimed to comprehensively evaluate multidimensional clinical information relevant to the pathophysiology of Acute Pulmonary Embolism (APE). All predictor variables were collected within 24 h before or after the CTPA examination, and included: Demographic characteristics: Sex, age, days of hospitalization (DAYS). Blood gas and oxygenation parameters: pH, partial pressure of arterial carbon dioxide (PCO₂), partial pressure of arterial oxygen (PO_2_), actual bicarbonate (HCO₃act), standard bicarbonate (HCO₃std), Base Excess (BE), arterial oxygen saturation (O₂sat), Total Carbon dioxide content (TCO_2_). Myocardial and metabolic markers: Creatine Kinase-MB (CK-MB), high-sensitivity cardiac Troponin T (cTnThs), Myoglobin (MYO), N-Terminal pro-B-type Natriuretic Peptide (NT-proBNP), C-Reactive Protein (CRP), Procalcitonin (PCT), Glucose (GLU), Lactate (Lac), adenosine deaminase (ADA), non-esterified fatty acids (NEFA), beta-2 Microglobulin (β2MG). Renal function and electrolytes: Creatinine (Crea), Uric Acid (UA), estimated Glomerular Filtration Rate (eGFR), Cystatin C (CysC), Sodium (Na), Potassium (K), Chloride (Cl), Calcium (Ca), Magnesium (Mg), inorganic Phosphorus (IP). Hepatic and nutritional indicators: Total Bilirubin (TBIL), Direct Bilirubin (DBIL), Alanine Aminotransferase (ALT), Aspartate Aminotransferase (AST), Gamma-Glutamyl Transferase (GGT), Total Protein (TP), Albumin (ALB), Albumin-to-Globulin ratio (AG), prealbumin (PA), Cholinesterase (CHE), high-density lipoprotein Cholesterol (HDLc). Coagulation and thrombotic markers: D-dimer (DDimer), Fibrinogen (FIBC), Thrombin Time (TT), Thrombin Time Ratio (TTR), Activated Partial Thromboplastin Time (APTT), APTT Ratio (APTTR), Prothrombin Time (PT), Prothrombin Time activity (PT%), International Normalized Ratio (INR), Antithrombin (AT), Total Bile Acid (TBA), fibrin(ogen) degradation products (FDP). Tumor markers: Carbohydrate Antigen 199 (CA199), Carbohydrate Antigen 724 (CA724), Cytokeratin 19 Fragment (CYFRA21– 1), Neuron-Specific Enolase (NSE), Carcinoembryonic Antigen (CEA), Alpha-Fetoprotein (AFP). Complete blood count analysis: Red Blood Cell count (RBC), Hemoglobin (HGB), Hematocrit (HCT), Mean Corpuscular Volume (MCV), Mean Corpuscular Hemoglobin (MCH), Mean Corpuscular Hemoglobin Concentration (MCHC), Red Cell Distribution Width Coefficient of Variation (RDW-CV), White Blood Cell Count (WBC), Neutrophil Percentage (NEU), Lymphocyte percentage (Lymph), Monocyte percentage (MON), Platelet count (PLT), Mean Platelet Volume (MPV). Additional enzymatic markers: Alpha-hydroxybutyrate Dehydrogenase (aHBDH), Lactate Dehydrogenase (LDH), Creatine Kinase (CK). All measurements were performed according to the standard operating procedures of the hospital's clinical laboratory, using International System of Units (SI units).

### Development, evaluation, and interpretability analysis of the prediction model

Building upon the features selected by LASSO, we adopted a multi-model ensemble strategy to develop and evaluate an Acute Pulmonary Embolism (APE) prediction model, followed by interpretability analysis of the optimal model. The specific workflow was as follows: 1) Handling of Missing Data: Variables with missing values were imputed using multiple imputation (R software, version 4.1.2, mice package). 2) Feature Selection: LASSO (Least Absolute Shrinkage and Selection Operator) regression was performed on all independent variables using R software (version 4.1.2; glmnet package). The optimal regularization parameter (λ) was determined via 10-fold cross-validation, identifying variables with non-zero coefficients. Subsequently, these LASSO-selected variables were subjected to multivariate logistic regression analysis (SPSS 25.0) to retain statistically significant independent predictors (p < 0.05) for model development. 3) Data Partitioning: Using stratified random sampling (Python, scikit-learn 1.1.3), all cases were randomly split into a training set (n = 454) and an independent test set (n = 195) at a 7:3 ratio. This ensured consistent APE-positive rate distribution between the two sets, facilitating evaluation of model generalizability. 4) Multi-Model Comparison and Selection: Eight classification models were constructed and optimized on the training set: eXtreme Gradient Boosting (XGBoost), Logistic Regression, Light Gradient Boosting Machine (LightGBM), Random Forest, Adaptive Boosting (AdaBoost), Gaussian Naive Bayes, Multi-layer Perceptron (MLP), and K-Nearest Neighbors (KNN). All models were implemented in Python (scikit-learn 1.1.3, xgboost 2.0.1, lightgbm 3.2.1). Model performance was assessed via 10-fold cross-validation, with the optimal model selected based on the Area Under the Receiver operating Characteristic Curve (AUC-ROC) on the test set.^13^ Calibration curves and Precision-Recall (PR) curves were also generated to evaluate the accuracy of predicted probabilities and classification performance on imbalanced data, respectively.^14^ 5) Validation and Stability Assessment of the Optimal Model: The selected optimal model (Logistic Regression) was trained using 10-fold cross-validation and validated on the independent test set. Learning curves (scikit-learn) were plotted to analyze loss variations between the training and validation sets, assessing overfitting or underfitting and ensuring model stability and reliability. 6) Model Interpretability Analysis: The SHAP (SHapley Additive exPlanations) framework (shap 0.43.0) was applied to the optimal model for global and local interpretation. SHAP summary plots were used to illustrate the overall direction and magnitude of each feature's contribution to predictions.^15^ For individual samples, SHAP force plots were generated to visually represent the specific impact of each feature on the predicted risk, thereby providing transparent and intuitive decision-making support and enhancing the model's credibility and utility in clinical practice.

### Statistical analysis

Baseline characteristics of all included variables were compared between the training and test sets. Continuous variables were presented as Median (first Quartile, third Quartile) [M (Q₁, Q₃)], with between-group differences assessed using the Mann-Whitney *U test*. Categorical variables were summarized as frequency (percentage) [n (%)], and group comparisons were performed using the Chi-Square test or Fisher's exact test (when the expected frequency was < 5). All statistical tests were two-sided, with a significance level set at p < 0.05. The analyses were primarily conducted using SPSS 25.0, R 3.6.1, and Python 3.4.3.

## Results

### Clinical characteristics of the study population

A systematic comparison of baseline characteristics was performed between the training (n = 454) and test (n = 195) sets. Statistical analysis revealed no significant differences in any of the included clinical or demographic variables between the two groups (all p > 0.05). This encompassed all key predictors, such as age, sex, blood gas parameters, myocardial injury markers, and coagulation profiles. These results indicate balanced baseline characteristic distributions between the training and test sets, ensuring data distribution consistency during model development and validation, and providing a reliable foundation for evaluating the training efficacy and generalizability of subsequent machine learning models (Table 1).

**Table 1:** Baseline characteristics in training cohort and testing cohort.

Variable	Training set (n = 454)	Testing set (n = 195)	*Z*	p
Diagnosis, n (%)				
No	247 (54.405)	94 (48.205)	2.103	0.147
Yes	207 (45.595)	101 (51.795)		
SEX, n (%)				
Female	196 (43.172)	81 (41.538)	0.149	0.700
Male	258 (56.828)	114 (58.462)		
AGE, median [IQR]	67.000 [55.000, 74.000]	68.000 [57.000, 74.000]	−0.917	0.359
pH, median [IQR]	7.425 [7.405, 7.446]	7.430 [7.412, 7.454]	−1.849	0.065
PCO2, median [IQR]	38.620 [35.700, 41.260]	38.410 [35.500, 41.120]	0.630	0.529
PO2, median [IQR]	79.190 [70.370, 90.110]	78.600 [68.600, 89.440]	0.553	0.581
HCO3act, median [IQR]	24.900 [23.510, 25.970]	24.860 [23.200, 26.380]	−0.297	0.767
HCO3std, median [IQR]	24.790 [23.900, 25.900]	24.970[23.820, 26.070]	−0.978	0.328
BE, median [IQR]	0.400 [−0.500, 1.600]	0.490 [−0.600, 1.760]	−0.654	0.513
O2sat, median [IQR]	95.400 [93.200, 96.900]	95.330 [93.000, 96.760]	−0.005	0.996
TCO2, median [IQR]	25.700 [23.700, 27.700]	25.800 [23.500, 28.000]	−0.389	0.697
TBA, median [IQR]	5.180 [3.230, 8.300]	5.370 [3.400, 8.790]	−0.489	0.625
CKMB, median [IQR]	14.000 [10.000, 19.000]	13.400 [9.000, 18.000]	1.498	0.134
ADA, median [IQR]	11.000 [8.100, 14.000]	10.600 [9.000, 13.000]	0.602	0.547
Lac, median [IQR]	2.360 [1.960, 2.890]	2.374 [1.950, 3.030]	−0.220	0.826
NEFA, median [IQR]	0.504 [0.396, 0.623]	0.466 [0.394, 0.608]	1.632	0.103
GLU, median [IQR]	6.152 [5.390, 7.406]	6.240 [5.450, 7.583]	−0.642	0.521
eGFR, median [IQR]	113.100 [94.822, 134.746]	112.100 [93.560, 132.141]	0.727	0.468
Ca, median [IQR]	2.200 [2.110, 2.300]	2.210[2.130, 2.300]	−0.714	0.475
Mg, median [IQR]	0.850 [0.799, 0.900]	0.850[0.800, 0.900]	0.097	0.923
IP, median [IQR]	1.020 [0.910, 1.140]	1.030[0.910, 1.130]	−0.178	0.859
HDLc, median [IQR]	1.128 [0.941, 1.300]	1.129[0.929, 1.329]	−0.248	0.804
CHE, mean (±SD)	6075.572±2007.432	5906.869±1916.194	0.993	0.321
PA, mean (±SD)	175.756±65.003	169.692±66.961	1.078	0.281
CysC, median [IQR]	1.029 [0.907, 1.230]	1.037[0.884, 1.220]	0.223	0.824
CK, median [IQR]	73.000 [50.000, 123.000]	75.000[49.000, 121.900]	−0.262	0.793
aHBDH, median [IQR]	188.000 [159.900, 230.900]	194.400 [161.800, 243.000]	−0.926	0.355
LDH, median [IQR]	238.000 [203.000, 291.800]	245.000[201.000, 303.400]	−0.636	0.525
CRP, median [IQR]	31.833 [11.300, 64.233]	30.900[12.790, 61.653]	−0.018	0.986
β2MG, median [IQR]	2.540 [1.980, 3.186]	2.527 [2.119, 3.180]	−0.703	0.482
ProBNP, median [IQR]	508.772 [170.900, 1436.000]	523.300 [173.300, 1486.000]	−0.627	0.531
MYO, median [IQR]	42.051 [26.740, 75.660]	44.380 [26.390, 74.886]	−0.117	0.907
cTnThs, median [IQR]	0.015 [0.008, 0.027]	0.014 [0.008, 0.025]	0.118	0.906
PCT, median [IQR]	0.242 [0.170, 0.408]	0.210 [0.137, 0.378]	1.906	0.057
FDP, median [IQR]	11.795 [4.720, 25.280]	12.470 [5.990, 28.190]	−0.814	0.416
AT, median [IQR]	90.000 [79.000, 101.200]	91.700 [82.000, 101.000]	−1.095	0.273
CA199, median [IQR]	14.875 [9.060, 44.113]	15.411 [8.320, 40.473]	−0.092	0.927
CA724, median [IQR]	10.258 [3.550, 23.694]	9.785 [4.163, 21.758]	0.232	0.817
CYFRA211, median [IQR]	4.900 [2.620, 11.820]	6.251 [2.930, 13.758]	−1.081	0.280
NSE, median [IQR]	17.004 [13.400, 22.736]	17.390 [13.160, 23.640]	−0.076	0.940
CEA, median [IQR]	4.170 [2.220, 15.660]	4.210 [2.275, 12.996]	−0.170	0.865
AFP, median [IQR]	2.805 [2.284, 3.360]	2.765 [2.180, 3.340]	1.114	0.265
DAYS, median [IQR]	9.000 [6.000, 14.000]	10.000 [7.000, 15.000]	−1.672	0.094
HCT, median [IQR]	36.700 [32.700, 41.200]	36.890 [32.400, 41.100]	0.095	0.925
HGB, median [IQR]	118.000 [104.000, 131.000]	117.000 [100.000, 131.000]	0.514	0.607
Lymph, median [IQR]	16.000 [10.600, 22.900]	15.600 [9.500, 22.300]	0.909	0.363
MCH, median [IQR]	30.300 [29.000, 31.800]	30.400 [28.900, 31.800]	0.075	0.941
MCHC, median [IQR]	319.000 [309.000, 327.000]	318.000 [308.000, 328.000]	0.310	0.756
MCV, median [IQR]	95.100 [91.500, 98.400]	95.100 [91.100, 98.800]	0.095	0.924
MON, median [IQR]	7.700 [5.900, 9.400]	7.800 [5.800, 9.800]	−0.504	0.614
MPV, median [IQR]	11.400 [10.600, 12.190]	11.300 [10.400, 12.200]	0.490	0.624
NEU, median [IQR]	73.000 [64.800, 80.400]	73.200 [65.900, 81.300]	−0.647	0.518
PLT, median [IQR]	187.000 [140.000, 257.300]	179.000 [137.000, 251.000]	0.400	0.689
RBC, median [IQR]	3.900 [3.430, 4.340]	3.898 [3.340, 4.240]	0.318	0.750
RDWCV, median [IQR]	13.800 [13.000, 15.000]	13.800 [12.900, 15.100]	0.008	0.994
WBC, median [IQR]	7.160 [5.620, 9.360]	7.220 [5.210, 9.010]	0.539	0.590
TBIL, median [IQR]	11.300 [7.600, 16.000]	11.000 [7.800, 14.459]	1.156	0.248
DBIL, median [IQR]	3.400 [2.390, 5.130]	3.380 [2.530, 4.740]	0.003	0.998
ALT, median [IQR]	23.600 [14.000, 38.000]	24.000 [15.000, 41.400]	−0.641	0.521
AST, median [IQR]	27.000 [19.000, 38.000]	27.000 [19.000, 38.000]	0.019	0.985
GGT, median [IQR]	37.000 [22.000, 65.000]	36.00 0[22.000, 78.000]	−0.451	0.652
Na, median [IQR]	138.800 [136.400, 140.700]	138.900 [136.400, 141.000]	−0.384	0.701
K, median [IQR]	3.880 [3.630, 4.150]	3.900 [3.616, 4.230]	−0.447	0.655
Cl, median [IQR]	103.200 [100.600, 106.000]	103.000 [99.800, 106.000]	0.503	0.615
TP, median [IQR]	64.050 [58.838, 69.400]	63.700 [57.890, 68.580]	1.295	0.195
ALB, median [IQR]	35.800 [31.400, 39.200]	34.900 [31.210, 38.200]	1.328	0.184
AG, median [IQR]	1.29 7[1.090, 1.500]	1.290 [1.040, 1.470]	0.944	0.345
Crea, median [IQR]	66.200 [54.200,83.500]	70.000 [58.700, 82.600]	−1.941	0.052
UA, median [IQR]	300.400 [228.000, 377.000]	298.000 [237.000, 382.000]	−0.483	0.629
DDimer, median [IQR]	1790.000 [580.000, 3444.000]	1812.000 [665.000, 3409.800]	−0.103	0.918
FIBC, median [IQR]	3.840 [2.930, 4.910]	4.010 [3.110, 5.130]	−1.106	0.269
TTR, median [IQR]	0.980 [0.900, 1.070]	0.960 [0.890, 1.060]	1.551	0.121
TT, median [IQR]	13.900 [12.800, 15.200]	14.000 [13.100, 15.400]	−1.277	0.202
APTT, median [IQR]	30.300 [27.900, 33.300]	30.700 [27.900, 33.500]	−0.929	0.353
APTTR, median [IQR]	0.960 [0.880, 1.070]	0.970 [0.880, 1.040]	0.021	0.984
PT, median [IQR]	11.900 [11.100, 13.100]	12.200 [10.900, 13.600]	−0.831	0.406
PT%, median [IQR]	93.00 0 [81.000, 105.000]	91.000 [77.000, 103.000]	1.001	0.317
INR, median [IQR]	1.040 [0.974, 1.150]	1.060 [0.970, 1.170]	−0.946	0.344

pH, potential of Hydrogen; PCO₂, Partial Pressure of Carbon Dioxide; PO₂, Partial Pressure of Oxygen; HCO₃act, Actual Bicarbonate; HCO₃std, Standard Bicarbonate; BE, Base Excess; O₂sat, Oxygen Saturation; TCO₂, Total Carbon Dioxide; CK-MB, Creatine Kinase-MB; cTnThs, High-sensitivity cardiac Troponin T; MYO, Myoglobin; ProBNP, N-terminal pro-B-type Natriuretic Peptide; CRP, C-Reactive Protein; PCT, Procalcitonin; GLU, Glucose; Lac, Lactic acid; ADA, Adenosine Deaminase; NEFA, Non-Esterified Fatty Acid; β2MG, Beta-2-Microglobulin; Crea, Creatinine; UA, Uric Acid; eGFR, Estimated Glomerular Filtration Rate; CysC, Cystatin C; Na, Sodium; K, Potassium; Cl, Chloride; Ca, Calcium; Mg, Magnesium; IP, Inorganic Phosphorus; TBIL, Total Bilirubin; DBIL, Direct Bilirubin; ALT, Alanine Aminotransferase; AST, Aspartate Aminotransferase; GGT, γ-Glutamyl Transferase; TP, Total Protein; ALB, Albumin; AG, Albumin-to-Globulin Ratio; PA, Prealbumin; CHE, Cholinesterase; TTR, Transthyretin; HDLc, High-Density Lipoprotein Cholesterol; DDimer, D-Dimer; FIBC, Fibrinogen; TT, Thrombin Time; APTT, Activated Partial Thromboplastin Time; APTTR, APTT Ratio; PT, Prothrombin Time; PT%, Prothrombin Time activity; INR, International Normalized Ratio; AT, Antithrombin; TBA, Total Bile Acid; FDP, Fibrin(ogen) Degradation Products; CA199, Carbohydrate Antigen 199; CA724, Carbohydrate Antigen-724; CYFRA211, Cytokeratin 19 Fragment; NSE, Neuron-Specific Enolase; CEA, Carcinoembryonic Antigen; AFP, Alpha-Fetoprotein; RBC, Red Blood Cell Count; HGB, Hemoglobin; HCT, Hematocrit; MCV, Mean Corpuscular Volume; MCH, Mean Corpuscular Hemoglobin; MCHC, Mean Corpuscular Hemoglobin Concentration; RDW-CV, Red Cell Distribution Width-Coefficient of Variation; WBC, White Blood Cell Count; NEU, Neutrophil percentage; Lymph, Lymphocyte percentage; MON, Monocyte percentage; PLT, Platelet count; MPV, Mean Platelet Volume; aHBDH, α-Hydroxybutyrate Dehydrogenase; LDH, Lactate Dehydrogenase; CK, Creatine Kinase.

### Feature selection: LASSO and multivariable logistic regression analysis

**Fig. 1:**
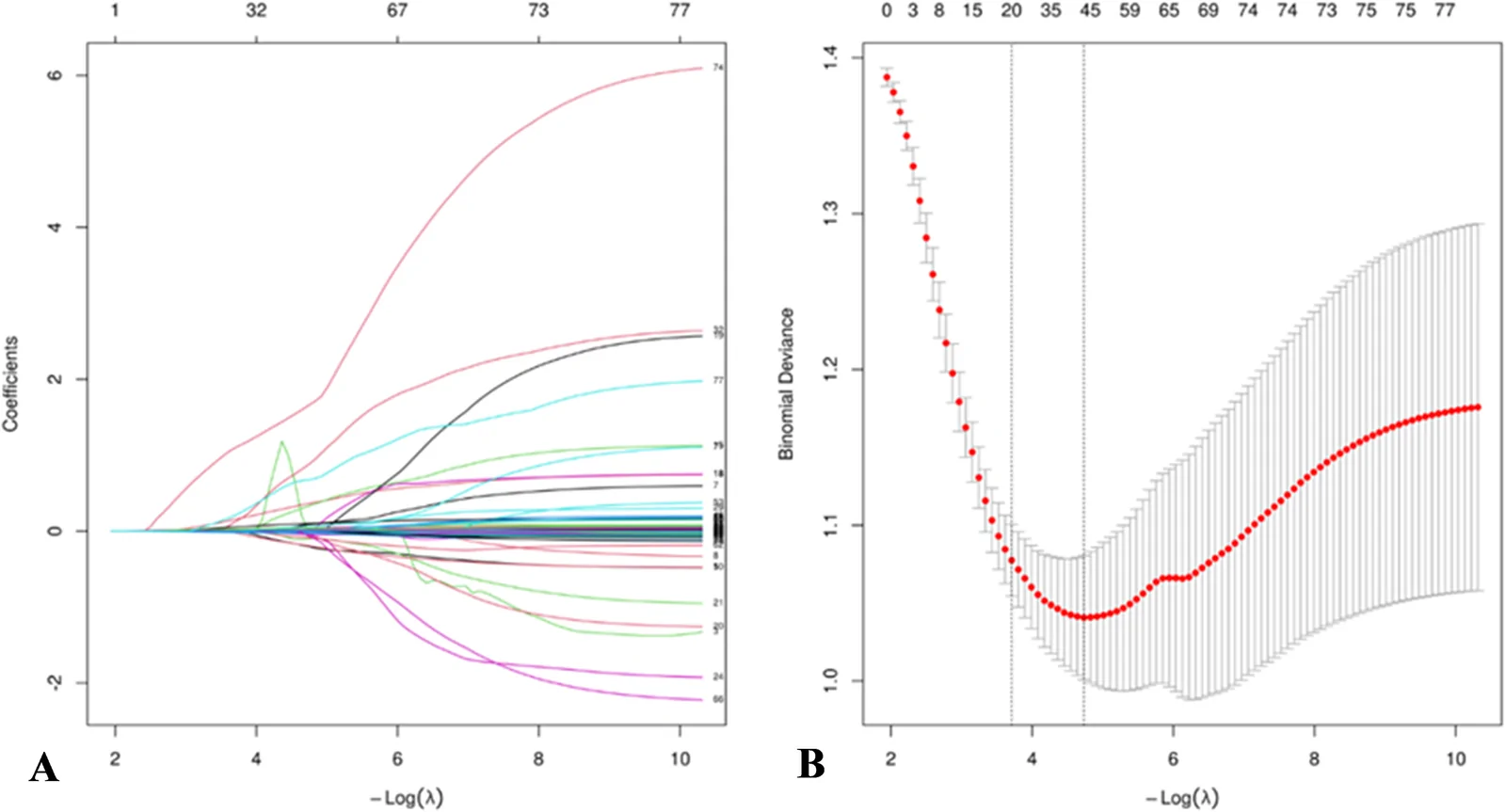
Feature selection using LASSO regression analysis. (A) Ten-fold cross-validation was employed to determine the optimal regularization parameter (lambda). The vertical line indicates the lambda value at which the model retained 20 non-zero coefficients. (B) Coefficient profiles of the 77 clinical features across a sequence of log(lambda) values in the LASSO model. The vertical dashed lines denote the lambda corresponding to the minimum mean squared error (λ = 0.009) and the lambda selected using the “one standard error” rule (λ = 0.024).

To identify the most relevant predictors for Acute Pulmonary Embolism (APE) diagnosis from the initially collected 77 clinical variables, this study employed Least Absolute Shrinkage and Selection Operator (LASSO) regression for feature dimensionality reduction (Fig. 1). The forest plot (Fig. S1) summarizes the effect estimates from the logistic regression model, illustrating the Odds Ratio and 95% Confidence Interval for each predictor variable. Through systematic adjustment of the regularization parameter λ, the optimal λ-value was determined as 0.024 based on the minimum distance standard error criterion (lambda.min). At this threshold, the model retained 20 non-zero coefficient predictors, including AGE, Lac, ProBNP, MYO, cTnThs, FDP, AT, CA199, Lymph, MCHC, MCV, MPV, PLT, WBC, TP, UA, TT, APTTR, PT, and INR. To further validate the independent predictive efficacy of these variables and control for potential confounding effects, these 20 indicators were subsequently subjected to multivariable logistic regression for secondary screening.^16^ Thirteen variables were ultimately identified as independent predictors for APE (all p < 0.05): AGE, Lac, FDP, AT, Lymph, MCHC, MCV, MPV, WBC, TP, PLT, UA, and APTTR (Table 2). This final feature set integrates demographic characteristics, metabolic indicators, coagulation function, inflammatory parameters, and hematological morphology information, thereby establishing a core variable foundation for subsequent development of a highly discriminative and clinically interpretable prediction model.

**Table 2:** Multivariate logistic regression analysis.

Variable	R	SE	OR (95% CI)	*Z*	p
(Intercept)	−1.713	3.403	0.180 (0.000 ‒ 139.092)	−0.503	0.615
AGE	0.045	0.009	1.046 (1.029 ‒ 1.065)	5.185	0.0
Lac	0.558	0.145	1.748 (1.326 ‒ 2.340)	3.842	0.0
ProBNP	0.0	0.0	1.000 (1.000 ‒ 1.000)	1.464	0.143
MYO	0.001	0.001	1.001 (1.000 ‒ 1.003)	1.611	0.107
cTnThs	0.75	0.737	2.118 (0.543 ‒ 11.224)	1.018	0.309
FDP	0.022	0.005	1.022 (1.013 ‒ 1.032)	4.515	0.0
AT	−0.02	0.006	0.981 (0.970 ‒ 0.991)	−3.459	0.001
CA199	−0.001	0.001	0.999 (0.997 ‒ 1.000)	−1.765	0.078
Lymph	0.054	0.012	1.055 (1.030 ‒ 1.082)	4.363	0.0
MCHC	−0.025	0.008	0.976 (0.961 ‒ 0.990)	−3.249	0.001
MCV	0.054	0.015	1.055 (1.025 ‒ 1.088)	3.573	0.0
MPV	−0.307	0.097	0.736 (0.606 ‒ 0.889)	−3.148	0.002
PLT	−0.003	0.001	0.997 (0.994 ‒ 1.000)	−2.001	0.045
WBC	0.133	0.038	1.142 (1.062 ‒ 1.232)	3.527	0.0
TP	−0.03	0.015	0.970 (0.942 ‒ 1.000)	−1.975	0.048
UA	0.003	0.001	1.003 (1.001 ‒ 1.005)	3.313	0.001
TT	0.044	0.039	1.045 (0.995 ‒ 1.133)	1.141	0.254
APTTR	2.01	0.614	7.461 (2.337 ‒ 26.027)	3.274	0.001
PT	0.032	0.072	1.032 (0.908 ‒ 1.200)	0.435	0.664
INR	0.993	0.906	2.699 (0.455 ‒ 15.709)	1.096	0.273

R, Regression coefficient; SE, Standard Error; OR, Odds Ratio.

### Comprehensive analysis of multiple classification models

**Fig. 2:**
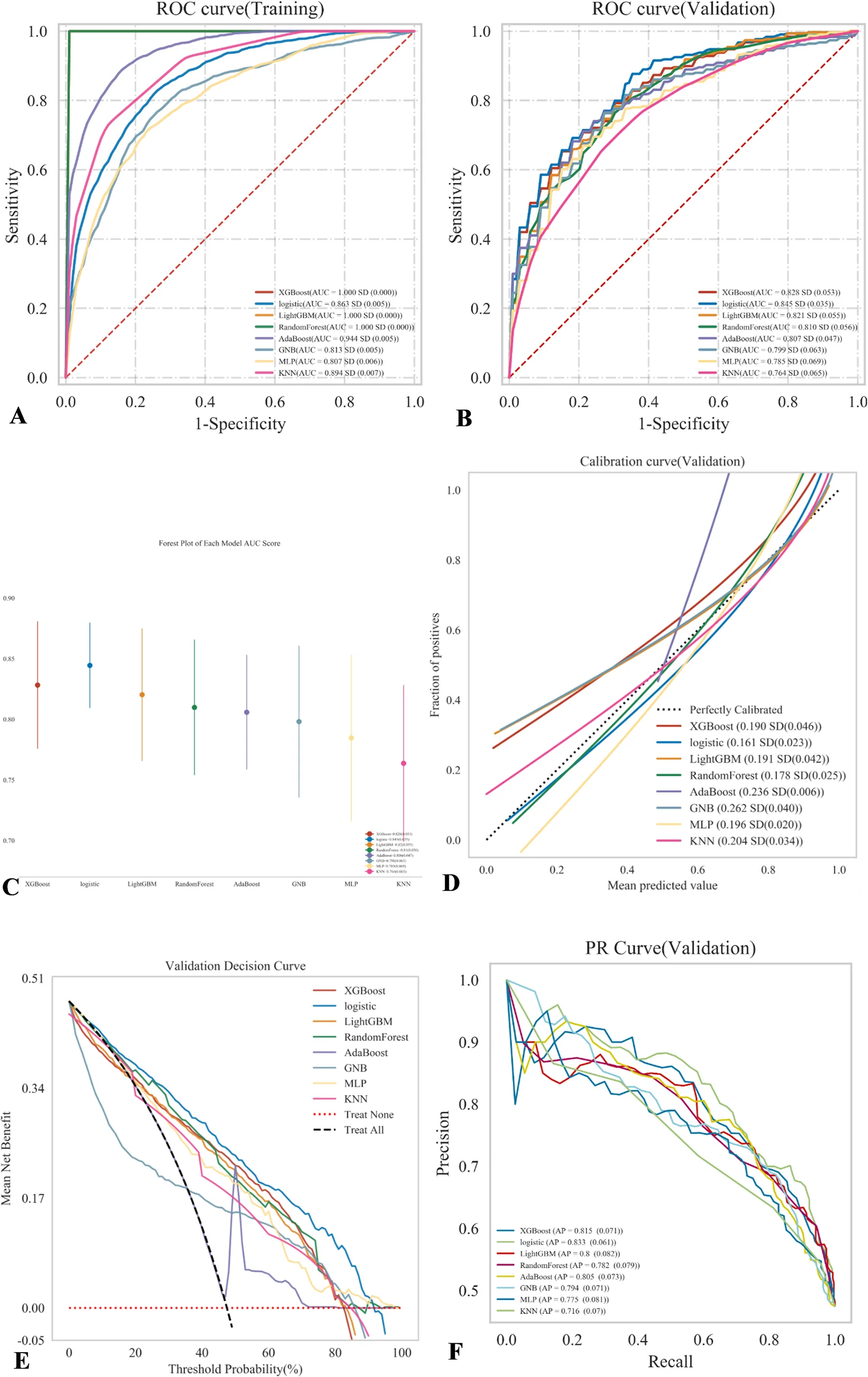
Comprehensive performance evaluation of machine learning models. (A) Receiver Operating Characteristic (ROC) Curves and Corresponding Area Under the Curve (AUC) values for each model on the training set. (B‒C) ROC curves and AUC values on the independent validation set. All models were evaluated on the same validation set to compare their generalizability. Models were assessed via ten-fold cross-validation on both the training and validation sets, with the figures displaying the average ROC curves across the ten validation runs. (D) Calibration curves for the validation set. The x-axis represents the predicted probability of a positive outcome, and the y-axis indicates the actual observed proportion of positive cases; the diagonal dashed line represents perfect calibration, and closer proximity of a model's fitted curve to this line indicates better calibration performance. (E) Decision Curve Analysis (DCA) for the validation set. The black dashed line denotes the strategy of “intervention for all patients”, the red dashed line denotes “intervention for no patients”, and the colored solid lines represent the clinical net benefit curves for each model. (F) Precision-Recall (PR) curves and Average Precision (AP) for the validation set. PR curves closer to the upper-right corner indicate superior model performance; higher AP values reflect better overall capability of the model to identify positive samples in imbalanced data. All performance metrics (AUC, AP, etc.) are presented as mean ± standard deviation across ten repeated experiments. Different models are distinguished by specific colors, with the corresponding associations detailed in the figure legend.

Eight machine learning models ‒ XGBoost, Logistic Regression, LightGBM, Random Forest, AdaBoost, Multilayer Perceptron (MLP), K-Nearest Neighbors (KNN), and Gaussian Naive Bayes (GNB) ‒ were systematically trained and evaluated on the training set. Preliminary analysis using the Area Under the receiver operating Characteristic Curve (AUC) indicated that XGBoost, LightGBM, and Random Forest demonstrated higher discriminative ability in the training set (higher training AUC; Fig. 2A). However, in the independent validation set, the Logistic Regression model achieved the highest AUC (Fig. 2B‒C), suggesting superior generalizability. Table 1S and Table 2S detail the baseline cohort characteristics and the comparative performance of multiple machine learning models in the training and validation sets, respectively. Given that AUC primarily reflects overall discriminative ability, calibration curves, Decision Curve Analysis (DCA), and precision-recall curves were further integrated to comprehensively assess the models’ clinical applicability and predictive reliability. Calibration curve evaluation revealed good agreement between the predicted probabilities of the Logistic Regression model and the observed event frequencies (Fig. 2D). Decision curve analysis demonstrated that the Logistic Regression model provided higher clinical net benefit across a wide range of threshold probabilities (Fig. 2E). In precision-recall curve analysis on the validation set, Logistic Regression also achieved the highest average precision value, indicating robust identification performance even in imbalanced datasets (Fig. 2F).

### Optimal model development and evaluation

**Fig. 3:**
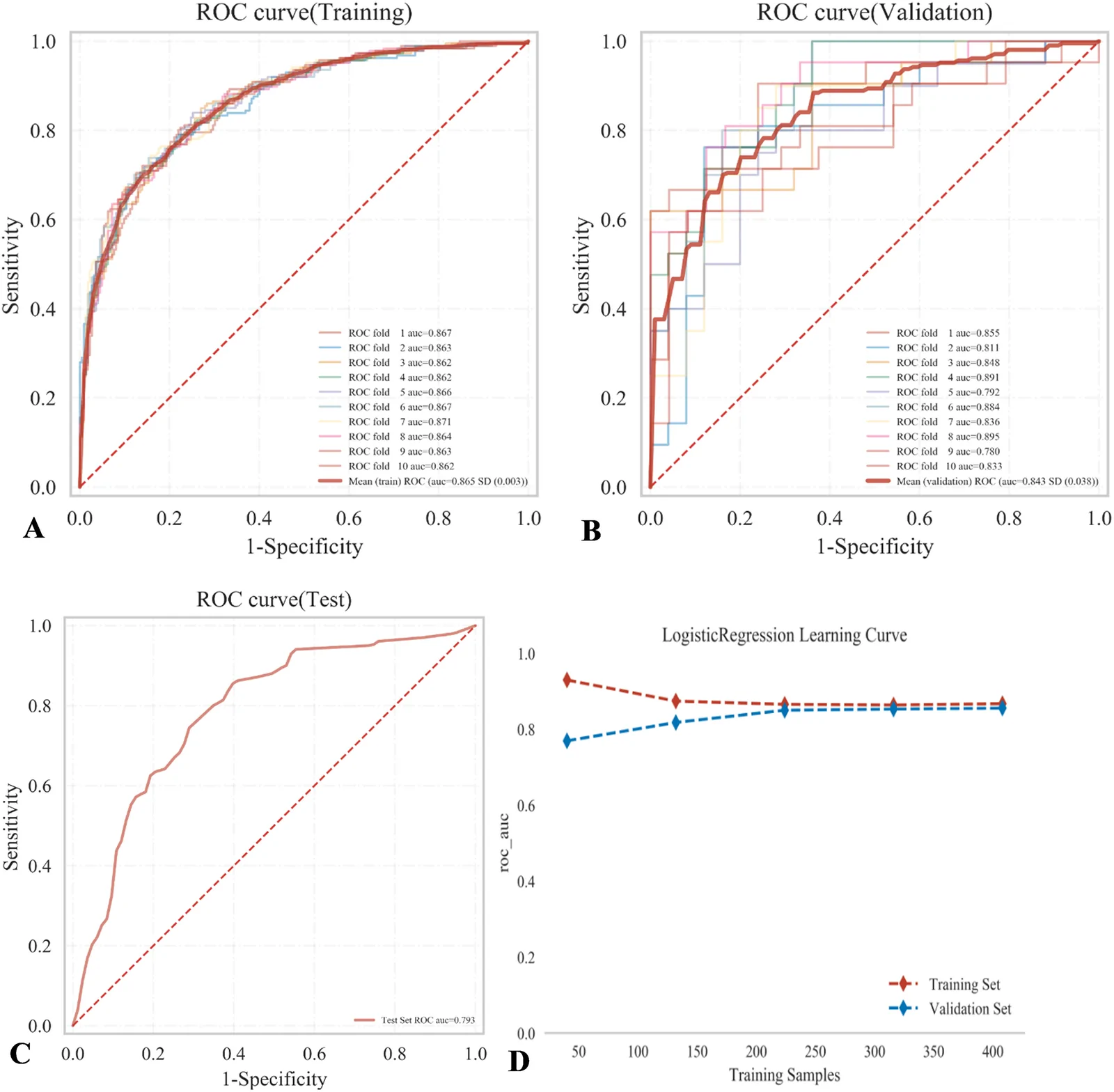
Training, Validation, and Testing Performance of the Logistic Regression Model. (A) Receiver Operating Characteristic (ROC) curve and corresponding Area Under the Curve (AUC) for the training set. The model was trained using the training set comprising 70% of the total samples and evaluated via ten-fold cross-validation; the figure displays the ROC curves from the ten cross-validation runs. (B) Mean ROC curve and AUC for the validation set during cross-validation. This reflects the stability of the model across different subsets of the training data. (C) ROC curve and AUC for the independent test set (comprising 30% of total samples), used to assess the final generalizability of the model. (D) Model learning curves. The red curve represents performance variation of the training set with increasing sample size, and the blue curve indicates corresponding performance changes in the validation set; the convergence trend of the two curves reflects the model's fitting status and stability. All AUC and accuracy values in the figure are presented as mean ± standard deviation (Mean ± SD) across ten repeated experiments.

The selected optimal Logistic Regression model was evaluated using ten-fold cross-validation. The results showed a mean AUC of 0.865±0.003 for the training set and 0.843±0.038 for the validation set. The model demonstrated stable performance in the independent test set, achieving an AUC of 0.793 and a classification accuracy of 0.718 (Fig 3A‒C). According to general model validation principles, when the performance on the validation set is not significantly better than that on the test set (typically with a difference of <10%), the model is considered not to exhibit substantial overfitting.^17^ The calibration plot (Fig. S2) demonstrated excellent agreement between the predicted probabilities and the observed outcomes across the entire risk spectrum in the validation cohort. Furthermore, the decision curve analysis (Fig. S3) revealed that the use of our model for clinical decision-making provided a superior net benefit compared to either the “treat-all” or “treat-none” strategies across a clinically relevant range of risk thresholds. In this study, the similar AUC values between the validation and test sets, along with the well-converged training-validation learning curve (Fig. 3D), indicate that this Logistic Regression model possesses robust generalizability and clinical applicability for the task of risk classification in acute pulmonary embolism.

### SHAP interpretability analysis: global feature contribution and individual risk prediction

**Fig. 4:**
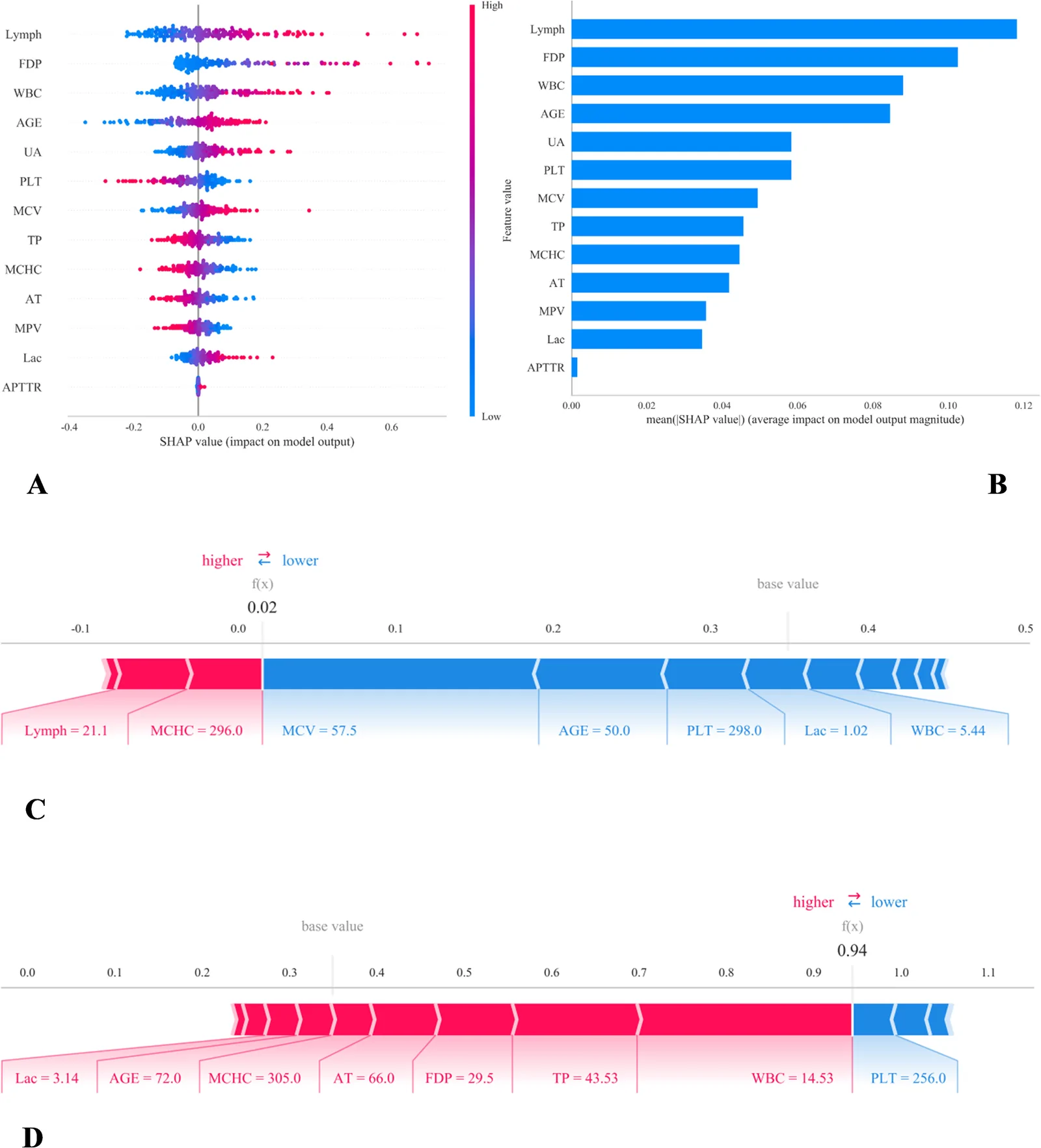
SHAP-based interpretability visualization of the model. (A) SHAP feature contribution bee swarm plot. Each vertical distribution band represents a predictive feature; the horizontal axis denotes SHAP values, indicating both the direction and magnitude of each feature's contribution to the model output (positive values shift predictions toward higher risk, negative values toward lower risk). Point colors reflect feature values (red for high, blue for low), illustrating monotonic or non-linear relationships between feature values and risk contribution. (B) Feature importance ranking based on mean absolute SHAP values. The bar chart quantifies the global contribution of each feature to model predictions, where bar length corresponds to the overall impact magnitude. (C) Low-risk case and (D) High-risk case SHAP decision decomposition plots. These panels separately display individualized prediction explanations for one APE-negative and one APE-positive patient. Arrow direction indicates the effect of a feature on the prediction (red arrows increase risk, blue arrows decrease risk), while arrow length reflects the magnitude of influence. The f(x) value shown at the bottom represents the model's final predicted probability for that sample. This visualization approach clearly reveals the decision path from multi-dimensional features to individualized risk assessment.

To elucidate the specific contribution of each clinical feature within the prediction model for Acute Pulmonary Embolism (APE) risk assessment, the SHAP (Shapley Additive exPlanations) framework was applied to the optimal model for interpretability analysis. As shown in Fig. 4A, the SHAP summary plot visually illustrates the nonlinear relationship between key features and predicted risk: elevated levels of Lymph, FDP, WBC, Age, UA, MCV, Lac, and APTTR, as well as decreased levels of PLT, TP, MCHC, AT, and MPV, significantly increased the model's tendency to predict positive cases. Fig. 4B further quantifies the global importance ranking of these features based on mean absolute SHAP values. Additionally, visual interpretation of two representative clinical cases (Fig. 4C–D) clearly demonstrates how the model generates differentiated predictions for low-risk and high-risk patients based on distinct feature combination patterns, thereby providing clinicians with an intuitive and reliable explainable basis for understanding the model's decision logic and assessing individualized risk.

## Discussions

This study successfully developed and validated a diagnostic model for predicting Acute Pulmonary Embolism (APE) risk in clinically suspected patients by integrating multi-dimensional clinical parameters and employing machine learning techniques. The model demonstrated excellent and stable performance via ten-fold cross-validation, achieving mean AUCs of 0.865±0.003 in the training set and 0.843±0.038 in the internal validation set. In the independent test set, the logistic regression model exhibited superior generalizability (AUC = 0.793), outperforming other complex models including XGBoost and Random Forest. This finding suggests that, within the context of our multi-dimensional clinical data, logistic regression may offer greater clinical utility and robustness compared to certain complex “black-box” models, attributable to its structural simplicity, reduced overfitting tendency, and interpretable coefficients.^18^^,^^19^

The model ultimately identified 13 key predictors spanning demographic characteristics (age), metabolic indicators (lactate, uric acid), coagulation markers (fibrin degradation products, activated partial thromboplastin time ratio, antithrombin), inflammatory parameters (white blood cell count, lymphocyte percentage), and hematological morphological indices (mean corpuscular volume, mean corpuscular hemoglobin concentration, mean platelet volume, platelet count, total protein). This feature ensemble comprehensively addresses multiple pathophysiological dimensions of APE: increased right ventricular afterload and myocardial injury (reflected by elevated lactate), thrombotic and fibrinolytic states (indicated by abnormal FDP, AT, and APTTR), systemic inflammatory response (suggested by WBC and Lymph variations), and secondary hemoconcentration with cellular morphological changes (demonstrated by MCV, MCHC, MPV, PLT, and TP alterations).^20^^,^^21^

Unlike traditional clinical prediction rules such as the Wells criteria or revised Geneva score, which primarily rely on symptoms, signs, and predisposing factors, ^5^ our model incorporates numerous objective laboratory parameters. To contextualize the added value of our model, we directly compared its performance against these established tools. At the optimally chosen threshold, our model demonstrated high sensitivity (90.3%vs. 81.2% for Wells) and comparable specificity (86.5%vs. 82.7% for Wells). Representative case examples further illustrate the model's performance: in a patient with atypical presentation and intermediate Wells probability, our model correctly identified high-risk status; in another with elevated D-dimer but low clinical probability, it appropriately ruled out PE, avoiding unnecessary CTPA. Particularly noteworthy is the identification of routine hematological indices ‒ Mean Corpuscular Hemoglobin Concentration (MCHC) and mean platelet volume (MPV) ‒ as independent predictors. While previous studies have predominantly associated these markers with cardiovascular diseases or inflammatory conditions,^22^^,^^23^ our findings confirm their incremental value in APE risk stratification, likely attributable to alterations in erythrocyte and platelet physiology induced by APE-related acute stress responses and microcirculatory changes. Information on anticoagulant use prior to CTPA was not systematically collected. However, in our institutional protocol, hemodynamically stable patients with suspected PE typically undergo CTPA before anticoagulation is initiated. Moreover, all predictor variables were restricted to data collected within 24-h prior to CTPA, a time window during which most patients had not yet received therapeutic anticoagulation. Therefore, the potential confounding effect of anticoagulation on model predictions is likely minimal. Nevertheless, external validation in cohorts with systematic anticoagulation records is warranted to confirm our findings. Furthermore, the inclusion of Uric Acid (UA) as a metabolic marker may reflect increased purine metabolism due to tissue hypoxia or the frequent comorbidity of metabolic syndrome in APE patients, with its association to adverse prognosis previously documented.^24^ It is noteworthy that D-dimer did not emerge as an independent predictor in our final model. LASSO regression, which performs variable selection among correlated predictors, likely subsumed the predictive signal of D-dimer into other retained coagulation parameters, particularly Fibrin Degradation Products (FDP) and Antithrombin (AT). FDP, like D-dimer, reflects plasmin-mediated fibrin degradation but may capture broader aspects of thrombotic burden. This finding does not diminish the clinical utility of D-dimer as an initial screening test; rather, it suggests that when a comprehensive panel of laboratory markers is available, the incremental value of D-dimer may be partially replaced by more specific or less redundant variables. These insights expand our understanding of APE-related biomarkers, suggesting that integrative analysis of multi-system routine laboratory parameters can more comprehensively characterize the pathophysiological state of patients.

A key innovation of this study is the systematic application of the SHAP framework to provide both global and individualized explanations for the optimal logistic regression model. SHAP analysis not only quantifies the overall importance of each feature (Fig. 4B) but more importantly, intuitively reveals complex relationships between feature values and predicted risk (Fig. 4A). For example, the analysis clearly demonstrates that elevated Lactate (Lac) and Fibrin Degradation Products (FDP) significantly contribute to increased risk prediction, aligning with their established roles as markers of tissue hypoperfusion and secondary hyperfibrinolysis, respectively, with clear clinical significance.^25^^,^^26^ For indicators such as Platelet count (PLT), SHAP plots reveal potential nonlinear or threshold effects in their association with risk, which are difficult to visualize through traditional logistic regression coefficients alone. By presenting SHAP decision breakdown plots for typical high- and low-risk cases (Fig. 4C–D), we visually illustrate for clinicians how the model integrates multiple abnormal indicators to calculate individualized risk probabilities. This “white-box” interpretation significantly enhances the model's credibility and clinical acceptability, enabling physicians to understand and prudently incorporate its decision-support recommendations ‒ a critical step toward transitioning AI-assisted diagnosis from “performance validation” to “clinical implementation”.

The findings of this study demonstrate clear potential for clinical translation. Against a backdrop of increasing concerns over strained healthcare resources and medical overuse,^27^ this model could serve as an effective triage tool prior to CTPA. For patients predicted to be low-risk, clinicians may be more confident in deferring or avoiding invasive and radiation-exposing CTPA, opting instead for alternative diagnostic considerations or short-term close monitoring. This approach could reduce unnecessary radiation exposure and contrast-induced nephropathy while optimizing resource allocation. For patients predicted to be high-risk, the model reinforces the urgency of confirmatory testing, potentially shortening time to diagnosis and facilitating earlier treatment initiation. The proposed model can be readily integrated into existing clinical workflows. All required laboratory tests are routinely ordered for patients with suspected acute pulmonary embolism. The model output can be automatically calculated once laboratory results are available, either through integration with the electronic medical record system or via a simple web-based calculator. Importantly, the model does not require any additional testing beyond standard care, and its computational cost is negligible.

Several limitations must be acknowledged. First, this was a single-center retrospective study with a relatively limited sample size (n = 649). Although internal validation demonstrated good stability, external validation in larger, multicenter, prospective cohorts is needed to confirm generalizability. Second, despite including numerous variables, some potentially important clinical information, such as detailed symptom descriptions, dynamic vital sign trends, electrocardiographic features, and lower extremity venous ultrasound results, may have been omitted. Furthermore, our model is intended only for hemodynamically stable patients. it should not delay emergent treatment in unstable cases, where immediate reperfusion therapy is indicated regardless of diagnostic testing. Future research could incorporate natural language processing to extract symptom-related text features or integrate time-series data analytics to further enhance model performance. Finally, this study has not yet evaluated the model within a real-world clinical workflow through prospective interventional trials. Whether model implementation ultimately improves patient outcomes, such as reducing mortality or decreasing unnecessary CTPA rates, remains to be investigated and represents an important direction for future research.

## Conclusions

This study successfully developed and validated an acute pulmonary embolism risk prediction model utilizing a machine learning framework integrated with SHAP interpretability. By synthesizing multidimensional clinical indicators and employing logistic regression as the core algorithm, the model demonstrated robust generalizability in an independent test set. The individualized risk interpretation generated by the model facilitates clinical decision-making and can serve as an effective auxiliary tool for optimizing CTPA examination strategies, thereby providing a novel approach toward achieving precision diagnostics and treatment.

## Ethical and reporting statement

This study was conducted in accordance with the Strengthening the Reporting of Observational Studies in Epidemiology (STROBE) guidelines for cross-sectional studies. The completed STROBE checklist is provided as Supplementary Material.

### Ethical approval and consent to participate

This retrospective study was conducted in accordance with the ethical principles of the Declaration of Helsinki. This study received ethical approval from the Institutional Ethics Review Committee of Dazhou Central Hospital. (Approval n°20,260,109). Informed consent was waived by the same committee because the study used fully anonymized historical patient data.

### Authors’ contributions

Q.L: Conceptualization; methodology; investigation; data curation; formal analysis; writing-original draft preparation; writing-review & editing.

H.Q: Conceptualization; Methodology. investigation; formal analysis; writing-review & editing; software.

J.X: Formal analysis; Project administration; resources; supervision; validation; writing-review and editing.

All authors read and approved the final manuscript.

### Funding

This research received no specific grant from any funding agency in the public, commercial, or not-for-profit sectors.

## Declaration of competing interest

The authors declare no conflicts of interest.
